# *Eureiandra orientalis* (Cucurbitaceae), a new species from Kenya and Tanzania

**DOI:** 10.3897/phytokeys.280.206680

**Published:** 2026-10-15

**Authors:** Ruo-Zhou Wang, Zhao-Hui Ran, Hanno Schaefer, Quentin Luke, Neduvoto Piniel Mollel, Paul Muigai Kirika, Christopher Chapano, Xue Yan, Sheng-Wei Wang, Neng Wei, Qing-Feng Wang

**Affiliations:** 1 State Key Laboratory of Plant Diversity and Specialty Crops, Wuhan Botanical Garden, Chinese Academy of Sciences, Wuhan 430074, Hubei, China State Key Laboratory of Plant Diversity and Specialty Crops, Wuhan Botanical Garden, Chinese Academy of Sciences Wuhan China; 2 College of Horticulture and Gardening, Heilongjiang Bayi Agricultural University, Daqing 163319, Heilongjiang, China College of Horticulture and Gardening, Heilongjiang Bayi Agricultural University Daqing China; 3 University of Chinese Academy of Sciences, Beijing 100049, China University of Chinese Academy of Sciences Beijing China; 4 Plant Biodiversity, Department of Life Science Systems, Technical University of Munich (TUM), Emil-Ramann-Str. 2, D-85354 Freising, Germany Plant Biodiversity, Department of Life Science Systems, Technical University of Munich (TUM) Freising Germany; 5 East African Herbarium, National Museums of Kenya, P.O. Box 45166-00100, Nairobi, Kenya East African Herbarium, National Museums of Kenya Nairobi Kenya; 6 Tanzania Plant Health and Pesticides Authority, National Herbarium of Tanzania, P.O. Box 3024, Arusha, Tanzania Tanzania Plant Health and Pesticides Authority, National Herbarium of Tanzania Arusha Tanzania; 7 National Herbarium and Botanic Garden, Fifth Street Extension, Alexandra Park, P.O. Box A889, Avondale, Harare, Zimbabwe National Herbarium and Botanic Garden Harare Zimbabwe; 8 Sino-Africa Joint Research Center (SAJOREC), Chinese Academy of Sciences, Wuhan 430074, Hubei, China Sino-Africa Joint Research Center (SAJOREC), Chinese Academy of Sciences Wuhan China

**Keywords:** Coastal forests of Eastern Africa, East Africa, *
Eureiandra
*, new species, taxonomy

## Abstract

*Eureiandra orientalis*, a new species of Cucurbitaceae from Kenya and Tanzania, is described and illustrated. It was previously treated as *Eureiandra* sp. A by Charles Jeffrey in the "Flora of Tropical East Africa", and was later referred to as *Eureiandra orientalis* Q.Luke ined., but it has never been formally described and named. Morphologically, the new species is most similar to *E. fasciculata*, but is readily distinguished from other members of *Eureiandra* by its nearly glabrous habit, with hairs mainly restricted to the nodes, leaf veins and margins, dark-green glossy leaves, denser shortly pedunculate male inflorescences, ovate petals, larger fruits, and oblong-elliptic blackish brown seeds with indistinct margins and fibrous testa. In addition, the two species differ in phenology: in *E. orientalis*, the leaves are fully developed at flowering, whereas in *E. fasciculata*, the stems are leafless at anthesis. The new species is mostly known from coastal forests of Eastern Africa in Kenya and Tanzania, one of the globally recognized biodiversity hotspots.

## Introduction

*Eureiandra* Hook.f. (Hooker 1867: 825) is a small African genus of Cucurbitaceae comprising eight species, distributed mainly in tropical and subtropical eastern and southern Africa, with *E. balfourii* Cogn. & Balf.f (Cogniaux and Balfour 1884: 404) restricted to the island of Socotra (Jeffrey 1967; Schaefer and Renner 2011; Schaefer 2020). In the current classification, the genus is placed in tribe Coniandreae of subfamily Cucurbitoideae (Schaefer et al. 2009; Schaefer and Renner 2011). It is characterized by simple tendrils, five free petals, broad anther connectives, beaked fruits, and dark, subglobose to ovate seeds, features that distinguish it from morphologically similar genera such as *Coccinia* Wight & Arn. (Wight and Arnott 1834: 347).

Although the economic or ethnobotanical significance of *Eureiandra* remains poorly understood, the roots of *E. balfourii* are reportedly used in traditional medicine on Socotra, and crude extracts have shown some biological activity in preliminary pharmacological studies (Ali et al. 2007; Mothana et al. 2009; Bakthira et al. 2011). However, these studies remain limited, and the genus has not received broader attention outside a limited number of taxonomic treatments.

In the framework of the "Flora of Kenya" project (Zhou et al. 2017; Wei et al. 2026), botanical explorations of the Kenyan coastal regions have been performed since 2015. During this fieldwork, plants of *Eureiandra* with nearly glabrous stems and leaves were collected. Detailed comparison with herbarium specimens housed at EA and K, together with a literature survey, showed that these plants match a taxon known from several collections from Kenya and Tanzania, which Jeffrey (1967) treated as “*Eureiandra* sp. A” in the "Flora of Tropical East Africa" (FTEA) without formally naming it. The taxon remained unnamed for decades, until Luke (2005), in his annotated checklist of the plants of Shimba Hills, referred to it as *Eureiandra orientalis* Q.Luke (ined.), but unfortunately without formally publishing this name. Here, we provide a formal description, diagnosis, and illustration of this East African species.

## Material and methods

Morphological characters are based on living plants observed in the field, as well as examination of dry herbarium specimens. A total of 56 specimens representing 41 unique collections of “*Eureiandra* sp. A” were examined (22 deposited at EA including the holotype, 24 deposited at K including the isotype, 6 deposited at MO, 3 deposited at HIB, and 1 deposited at WAG), while c. 140 digitized specimens of other *Eureiandra* species from K were studied online. Photographs of habitat and morphological features were taken with a Nikon D810 camera. Additional measurements were made from images of herbarium specimens using ImageJ software (https://imagej.net/ij/). Comparative assessment was based primarily on the morphology of the putative new species and its presumed closest relative, *Eureiandra fasciculata* (Cogn.) C.Jeffrey (1962: 353), as well as relevant taxonomic literature (Jeffrey 1967, 1978; Jeffrey and Thulin 1993; Newton and Njoroge 2023). All morphological data for *E. orientalis* were obtained from field measurements of living plants, examination of the 56 herbarium specimens and the FTEA description, whereas characters of *E. fasciculata* were taken from the FTEA description, Jeffrey (1967), and examination of available herbarium specimens. Additional taxonomic information was obtained from the Global Biodiversity Information Facility (GBIF, https://www.gbif.org/), JSTOR Global Plants (https://plants.jstor.org/), and Plants of the World Online (POWO, https://powo.science.kew.org/).

## Results

### Morphological comparison

Morphologically, *Eureiandra orientalis* is most similar to *E. fasciculata* in its annual herbaceous stems, palmately 3–5-lobed leaves, and female flowers with five staminodes. However, the two species differ consistently in several vegetative and reproductive characters (Table 1). *Eureiandra orientalis* has stems that are nearly glabrous, with sparse hairs mainly restricted to the nodes, whereas *E. fasciculata* has pubescent stems. Its leaves are larger (6.6–18.7 × 6.1–26.0 cm), darker green, and glossy, with hairs only confined to the veins and margins, while those of *E. fasciculata* are smaller (2.4–11.5 × 1.4–10.0 cm), paler, and rough or puberulous on the surface. The male flowers of *E. orientalis* are short pedunculate inflorescences (peduncle 4–5 mm long), whereas those of *E. fasciculata* are solitary or in fewer-flowered clusters lacking a distinct peduncle. In addition, *E. orientalis* has ovate petals, larger fruits (6.5–11.5 × 2.6–4.5 cm), and oblong-elliptic seeds with indistinct margins and fibrous testa. By contrast, *E. fasciculata* has obovate petals, smaller fruits (4.5–8.5 × 2.4–3.5 cm), and subglobose seeds with a distinct margin and smooth testa. The two species also differ in phenology: in *E. orientalis*, the leaves are fully developed at anthesis, whereas in *E. fasciculata*, the stems are often leafless during flowering.

**Table 1.** Comparison between *Eureiandra orientalis* and *E. fasciculata*.

**Character**	***E. orientalis***	***E. fasciculata***
Stem	Nearly glabrous, sparsely hairy at the nodes	Pubescent
Leaf blade	6.6–18.7 × 6.1–26.0 cm, dark green, glossy, hairy only on veins and margins	2.4–11.5 × 1.4–10.0 cm, bright green, rough or puberulous
Petiole	1.7–6.2 cm	1.0–3.0 cm
Male inflorescence	Solitary or in clusters of 2–14 flowers, shortly pedunculate	Solitary or in clusters of 2–6 flowers, peduncle absent
Petals	27–55 × 17–35 mm, ovate, pale orange with a darker center	18–35 × 14–20 mm, obovate, deep cream to orange-yellow, rarely white
Fruit	6.5–11.5 × 2.6–4.5 cm, ovoid-ellipsoid	4.5–8.5 × 2.4–3.5 cm, ellipsoid
Seeds	oblong-elliptic, c. 11 × 6 × 5.5 mm, indistinctly margined, slightly fibrous	subglobose, 6.5–10.2 × 6.0–7.5 × 4.5–6.2 mm, distinctly margined, smooth

### Taxonomic treatment

#### Eureiandra orientalis

Taxon classification

Plantae

CucurbitalesCucurbitaceae

N.Wei, Q.Luke, Kirika & Q.F.Wang sp. nov.

1911704F-8E80-5325-B36C-0341E3BC1E65

urn:lsid:ipni.org:names:77412420-1

Figs 1, 2, 3

= Eureiandra sp. A., Jeffrey, Fl. Trop. E. Africa, Cucurbit.: 41, 1967.

##### Type.

Kenya. Coast Province, Lamu District [now Lamu County], Witu, June 1957, *S. Rawlins EAH-11267* (holotype: EA!: isotype: K [K003452275]!).

**Figure 1.**
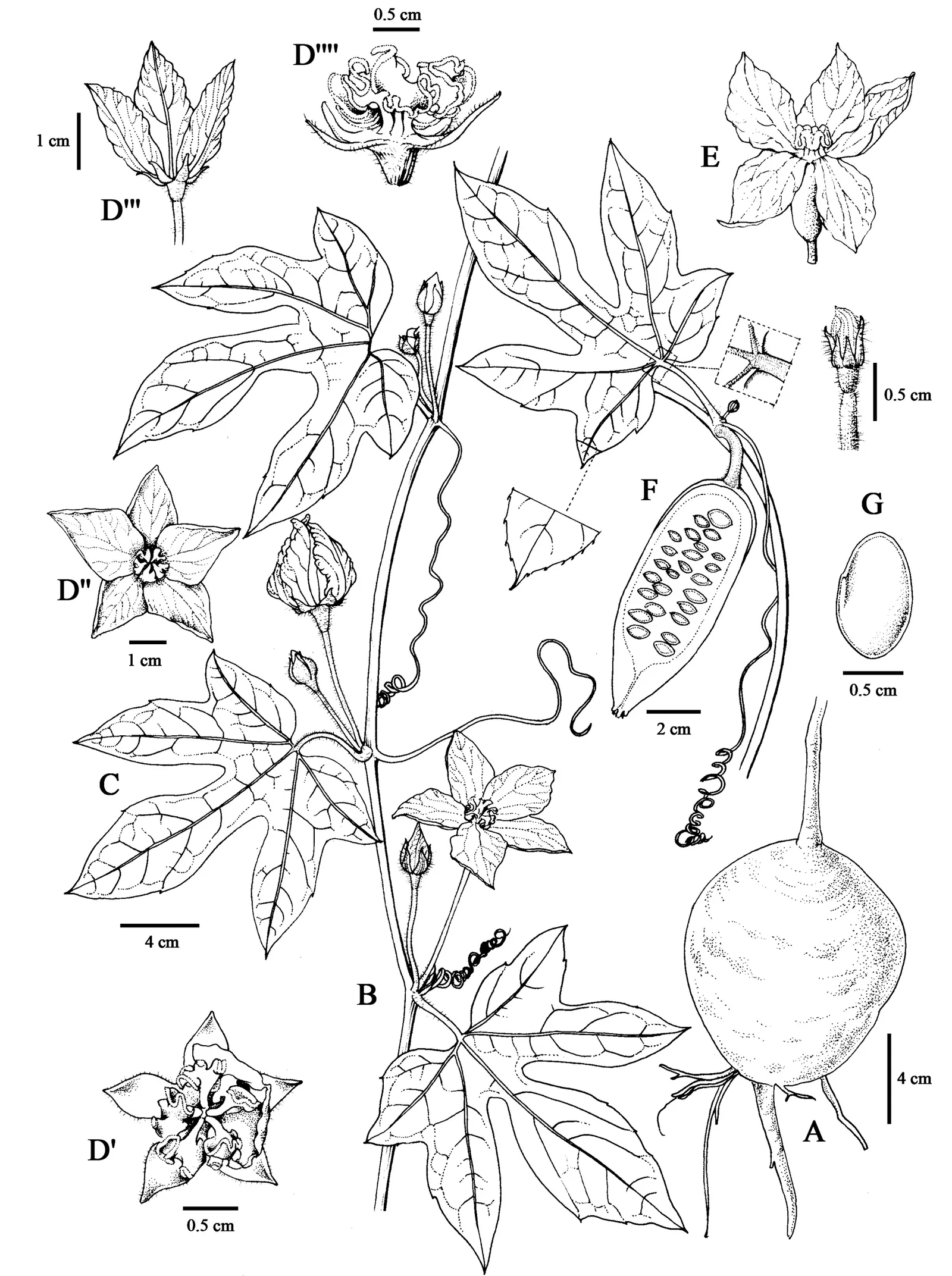
Illustration of *Eureiandra orientalis*. **A**. Tuber; **B**. Annual stem; **C**. Leaf; **D’**. Male flower with the corolla removed, apical view; **D”**. Male flower, apical view; **D”’**. Male flower, lateral view; **D”’’**. Male flower with the corolla removed, lateral view; **E**. Female flower, view from side; **F**. Longitudinally sectioned fruit, showing seeds; **G**. Seed. Drawn by Nan Jia.

**Figure 2.**
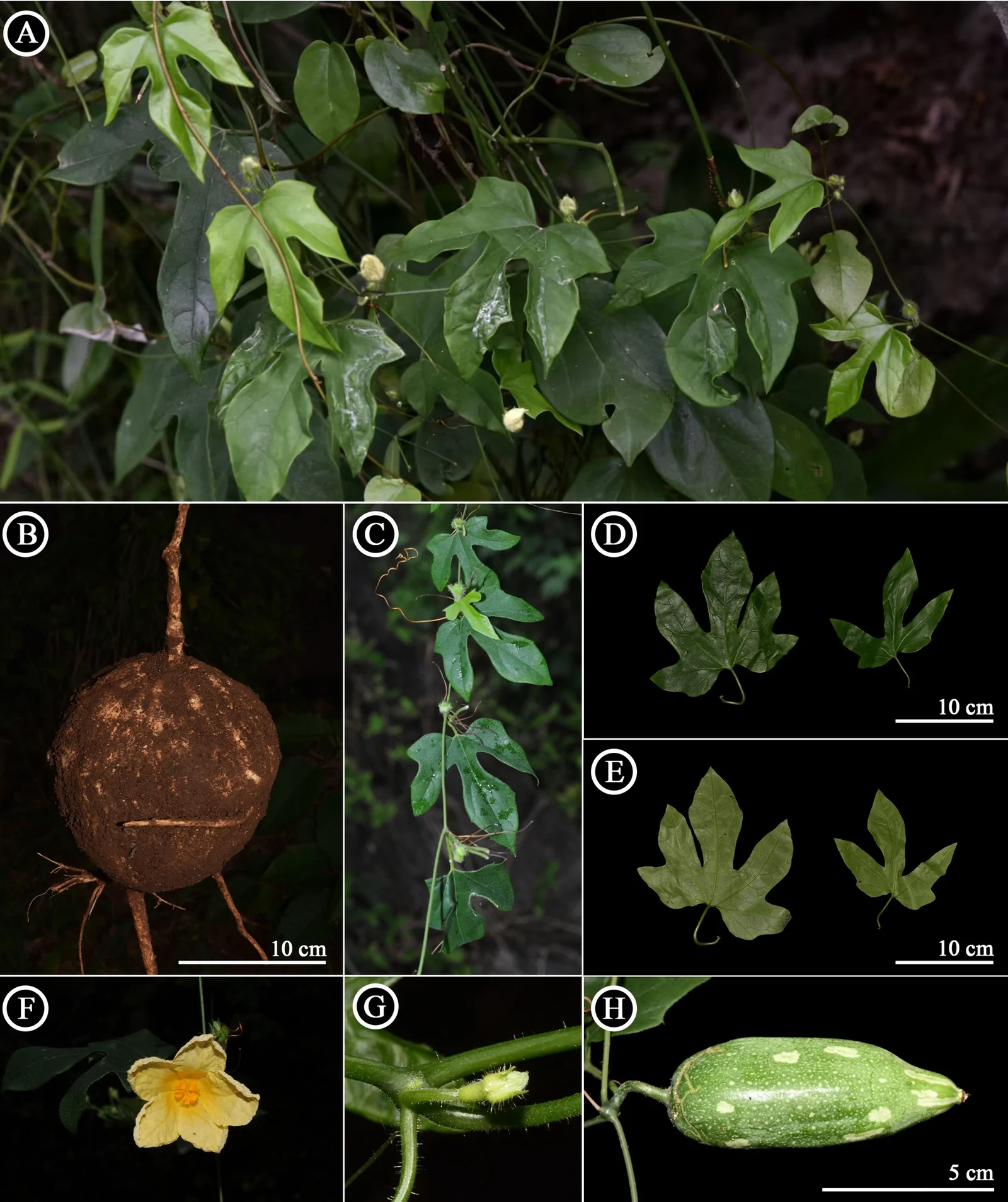
Habitat and morphological features of *E. orientalis*. **A**. Habitat, blooming on leafy vine; **B**. Tuber; **C**. Vine; **D**. Adaxial view of leaves; **E**. Abaxial view of leaves; **F**. Male flower; **G**. Immature female flower; **H**. Young fruit; **A, B, D, E, G, H**. Taken by Neng Wei; **C, F**. Taken by Veronicah Ngumbau.

**Figure 3.**
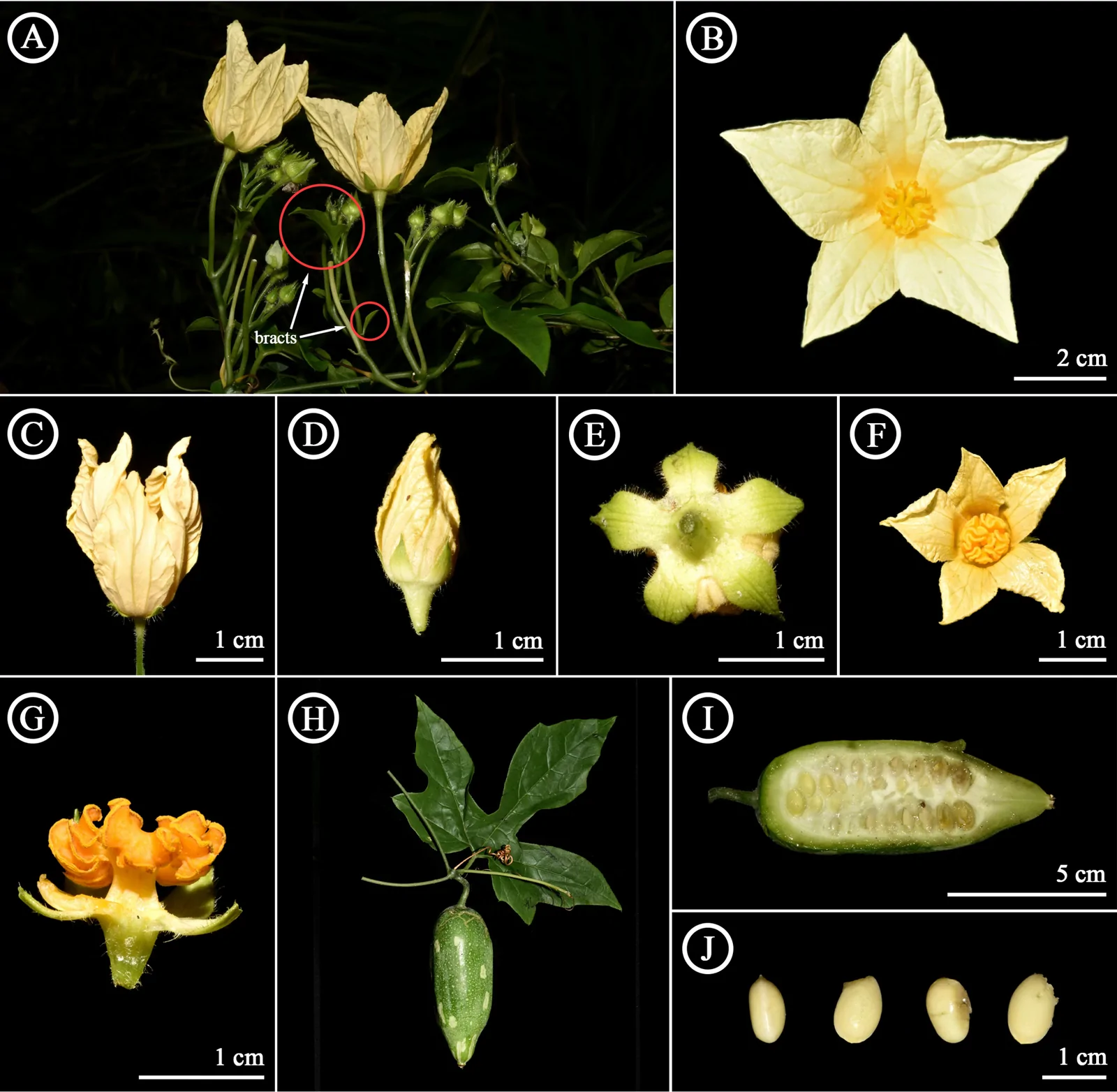
Reproductive structures of *E. orientalis*. **A**. Male inflorescence; **B**. Top view of male flower; **C**. Side view of male flower; **D**. Side view of immature male flower, showing receptacle; **E**. Bottom view of immature male flower, showing sepals; **F**. Top view of immature male flower, showing stamens; **G**. Close-up of mature stamens; **H**. Branch with fruit; **I**. Longitudinal section of fruit; **J**. Immature seeds. Photos by Neng Wei.

##### Diagnosis.

*Eureiandra orientalis* differs from the most similar *E. fasciculata* by its nearly glabrous stems with sparse hairs restricted to the nodes (vs. pubescent stems), by its dark-green glossy leaves with hairs mainly restricted to the veins and margins (vs. smaller, paler leaves that are rough or puberulous on the surface), by ovate petals (vs. obovate petals), by larger fruits (vs. smaller fruits), and by oblong-elliptic seeds with indistinct margin and slightly fibrous testa (vs. subglobose seeds with a distinct margin and smooth testa) (Table 1).

##### Description.

Climber to 4 m long, arising from a tuberous rootstock, this ellipsoid to subglobose, up to 17 cm in diam. ***Stems*** annual, herbaceous, glabrous, sparsely hairy at the nodes. ***Leaves*** simple, petiole 17–62 mm long, blade dark green and glossy, papery, becoming punctate, broadly ovate to reniform-cordate in outline, 66–187 × 61–260 mm, palmately 3–5-lobed, sparsely hairy on veins and margins, elsewhere glabrous, lobes narrowed towards the base, apex of lobes subacute and mucronulate, margins entire, undulate, or shallowly lobulate, the central lobe the largest. ***Male flowers*** solitary or in clusters of 2–14 on a short peduncle, 4–5 mm long, pedicels 12–72 mm long, sparsely pubescent. Bracts absent or occasionally 1–2, stalked, foliaceous, lanceolate, to 26 × 5 mm. Receptacle-tube obconical at base, abruptly widened beneath the lobes, 4–6 mm long, sepals triangular, 5–10 mm long, pale green, sparsely pubescent outside, entire, striate, spreading, apically recurved. Petals pale orange with darker centers, ovate, 27–55 × 17–35 mm, with 1–3 principal veins and numerous secondary veins; stamens 5, occasionally 3, inserted at the apex of the narrow part of the tube, filaments broad, anthers curved. ***Female flowers*** solitary or in pairs, ebracteate, pedicels 5–12 mm long, perianth similar to that of the male flowers. Ovary cylindrical to ellipsoid, slightly beaked, with numerous horizontally arranged ovules, floral tube short, staminodes 5, inserted near the apex of the tube, stigma 3-lobed. ***Fruit*** ovoid to ellipsoid, glabrous and fleshy, base rounded, apex gradually narrowed into a stout c. 15 mm beak, pale green with conspicuous white blotches, 6.5–11.5 × 2.6–4.5 cm, pedicel 1.4–2.2 cm long. ***Seeds*** oblong-elliptic, c. 11 × 6 × 5.5 mm, embedded in yellow pulp, blackish brown, with pale raphe and slightly fibrous testa; margins indistinct.

##### Phenology.

Flowering from January to August. Fruiting from May to October. East Africa has a bimodal rainfall regime (Pellikka et al. 2009). Flowering of *E. orientalis* coincides mainly with the coastal long rains (March–June) and adjacent months, and may also follow local rainfall at individual sites; it does not correspond to the short rains (October–December). Fruiting begins towards the end of the long rains and continues through the dry season.

##### Distribution and habitat.

**Figure 4.**
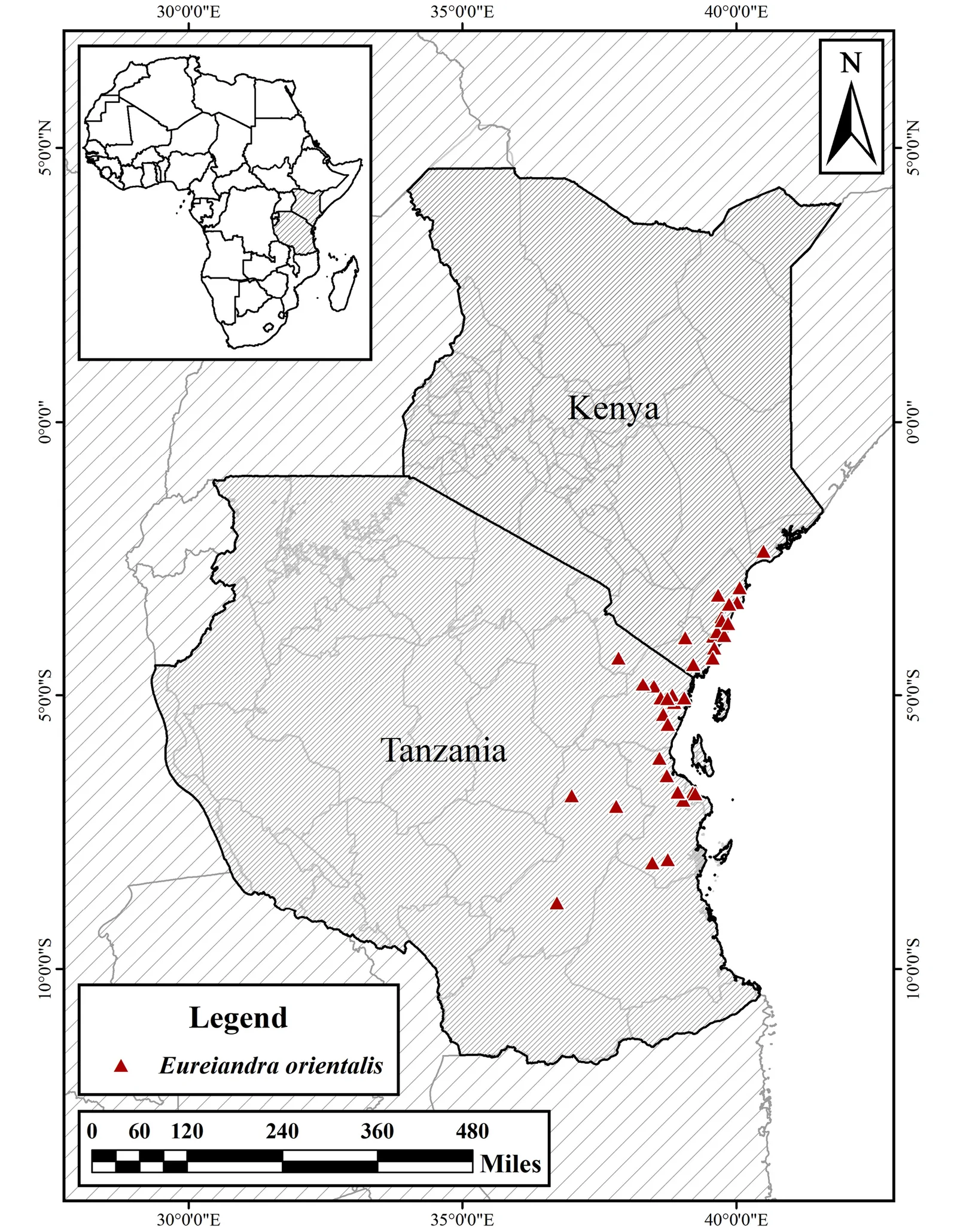
Distribution map of *Eureiandra orientalis*.

*Eureiandra orientalis* is currently known from the coastal lowlands of Kenya and Tanzania, with most records from Kilifi County in Kenya and coastal forest areas of Dar es Salaam and Tanga in Tanzania (Fig. 4). The species mainly occurs at low elevations between 0 and 760 m, commonly found on rocky limestone or coral rag slopes, at the edges of coastal forests, and in the understory of semi-deciduous forest.

##### Conservation status.

*Eureiandra orientalis* is currently known from multiple collections in coastal forests of Kenya and Tanzania. Given that *E. orientalis* is documented from 39 georeferenced occurrences in c. 15 locations, with an extent of occurrence (EOO) of c. 130,000 km^2^ and area of occupancy (AOO) of 148 km^2^, we assess the species as Least Concern (LC), following the IUCN Red List Categories and Criteria (IUCN 2012; IUCN Standards and Petitions Committee 2024). Coastal forest at several localities is already being lost to agricultural clearance and to quarrying of coral rag and limestone for cement. These activities are ongoing and are likely to reduce some populations in the near term. Remaining forest fragments therefore require prompt monitoring and protection, even though the species does not yet meet the thresholds for a threatened category.

##### Etymology.

The specific epithet ‘orientalis’ refers to the eastern African distribution of this species. The epithet follows the unpublished name used by Luke (2005).

##### Additional specimens examined (paratypes).

Kenya. Kilifi County: Ridge of red Magarini sands c. 3 miles west of Malindi, May 1959, *S. Rawlins 704* (EA!, K!) • Arabuko Forest, 8 km E of Jilore forest station, alt. 60 m, *Cynometra* forest with *Encephalartos hildebrandtii* on murram soil, 20 Jul 1972, *J.B. Gillett & S.P. Kibuwa 20040* (EA!, K!) • Gede Forest, Gedi National Monument, 3°18'S, 40°01'E, alt. 15 m, semi-deciduous lowland forest on coral rag with *Combretum schumannii*–*Gyrocarpus americanus*, 28 May 1985, *K. Gerhardt & M. Steiner 227* (EA!) • Kaya Kauma, 3°37'S, 39°44'E, alt. 120 m, edge of dry forest of *Brachylaena*, *Cynometra*, 8 Oct 1991, *Luke 2895A* (EA!, K!, MO!), *Luke 2895B* (EA!); Takaunga, small climber on edge of bush, flowers creamy yellow, 1961, *R.M. Graham D641* (K!); Mombasa to Takaunga, 1902, *A. Whyte s.n*. (K!) • Cha Simba Location, Cha Simba Rocks, 3°44.31'S, 39°41.70'E, alt. 199 m, forest on limestone outcrops, 3 Dec 2016, *V. Ngumbau & Mwadime N V0409* (EA!, HIB!) • Chasimba, 3°44'21.07"S, 39°41'40.79"E, alt. 214 m, rocky slope, limestone, 2 Jul 2024, *N. Wei KE-WN189* (HIB!) • Kilifi, 3°44'20.87"S, 39°41'40.76"E, alt. 214 m, rocky slope, limestone, 2 Jul 2024, *N. Wei KE-WN190* (HIB!) • Kikambala, sea level, 21 Jun 1956, *P.H. Irwin 261* (EA!, K!); Kilifi Forest, edge of forest along road, sea level, 11 Jun 1960, *P.H. Irwin 442* (EA!) • Jaribuni, alt. c. 150 m, woodland, 30 Jun 1984, *C.H.S. Kabuye*, *V.C. Gilbert & A. Robertson 84/91* (EA!) • Kaya Rabai, Kombeni river, 3°55'S, 39°35'E, alt. 90 m, rocky river through semi-deciduous forest, 22 May 1990, *Luke & Robertson 2272* (EA!) • Kaya Rabai, Bomu, 3°55'S, 39°35'E, alt. 100 m, semi-deciduous forest, 23 May 1990, *Luke & Robertson 2292* (EA!, K!). Kwale County: Jadini Beach, 18 miles south of Mombasa, open clearings in coastal forest near high water mark on coral rock, sea level, 26 Aug 1953, *R.B. Drummond & J.H. Hemsley 3992* (K!) • Dzombo, 4°26'S, 39°13'E, alt. 160 m, lowland forest, 4 Oct 1991, *Luke 2880* (EA!, K!). Tanzania. Tanga Region: • Usambara, 1893, *C. Holst 2380* (K!); W Usambara, Mombo–Wilhelmstal (Lushoto area), 22 May 1914, *A. Peter 56482* (K!) • E Usambara, lowland near Makumba, 10 Apr 1916, *A. Peter 55652* (K!) • E Usambara, Maramba, Mwele toward Tanga, steppe woodland, 18 Jun 1917, *A. Peter 56782* (K!) • Tanga, Amboni toward Tanga, near the warm sulphur springs, 16 Mar 1918, *A. Peter 56845* (K!) • Lushoto District, 29 Jun 1953, *R.B. Drummond & J.H. Hemsley 3069* (K, spirit) • Lushoto District, Kungului, Jul 1955, *C.D. Mgaga 52* (EA!, K!) • Mlingano, Sisal Research Station, 6 Feb 1964, *Yusuf Mohamed* s.n. (EA 13023) (EA!) • Pangani District, Msubugwe Forest Reserve, 15 Jan 1967, *H.M. Richards 21989* (K!) • Muheza District, Kwazennet village, 9 Jul 1971, *Sangiwa 129* (EA!); Pangani Falls Forest, 5°21'S, 38°40'E, alt. 150 m, 19 Feb 1993, *G.P. Clarke 3467* (K!) • Muheza District, Mringa Forest Reserve, 5°04'S, 38°45'E, alt. 250 m, 25 Feb 2008, *M.A. Mwangoka & T. Zuakuu 5645* (MO!) • Dar es Salaam Region: University College, Dar es Salaam, in a thicket, 8 Mar 1966, *L.B. Mwasumbi 10075* (EA!, K!) • Wazo Hill, 25 Apr 1968, *M. Batty 48* (K!) • Pwani Region: Bagamoyo District, Zaraninge Forest, 6°09'S, 38°36'E, alt. 100 m, 15 Mar 1990, *Frontier-Tanzania 1061* (K!, MO!) • Bagamoyo District, Kikoka Forest Reserve, Apr 1964, *S.R. Semsei 3847* (EA!, K!) • Kibaha, 40 km W of Dar es Salaam, 1 Jan 1971, *C. Flock 72* (K!) • Kibaha Centre, 40 km W of Dar es Salaam, 4 Feb 1972, *C. Flock 190* (MO!) • Pugu Hills, Mar 1939, *J.H. Vaughan 2773* (EA!), *J.H. Vaughan 2775* (EA!) • Rufiji District, 31 Dec 1931, *Amush 36* (EA!) • Morogoro Region: Kimboza Community Forest Reserve, 07°01'37"S, 037°48'31"E, alt. 240–360 m, 21 Mar 2006, *L. Festo, W.R.Q. Luke & C.J. Kayombo 2244* (EA!, MO!) • Ulanga District, Vigoi Division, Isongo Ward, Isongo Village, S of Mahenge town c. 12 km along road to Mwaya, Mzelezi Forest Reserve, along mountain ridge/escarpment, 08°47'21"S, 36°43'34"E, alt. 760 m, 12 Feb 2006, *W.J. Kindeketa & al. 2814* (MO!, WAG!).

##### Specimens examined for *Eureiandra fasciculata*.

Mozambique. Cabo Delgado Province • Quiterajo, along track from main camp at airstrip to Sacred forest (Nhanganemba), 11°48'13.7"S, 40°20'32.9"E, alt. 64 m, along track just below escarpment, in Boane forest, growing in shade, twining within undergrowth, leaves lobed, flower pale orange-yellow, 22 Nov 2009, *F. Crawford FC685* (K!) • Quirimbas National Park, pt 495–pt 496, coastal woodland, 12°46'S, 39°38'E, alt. 250 m, 9 Dec 2003, *W.R.Q. Luke, O.A. Kibure & E. Nacamo 9959* (MO!) • Nampula: Nova Chaves, junction of the roads to Mecubúri and Ribaué, c. 300–500 m towards Ribaué on a rocky mountain, 1983, *Alfredo Nuvunga 1234* (WAG!). Tete: Boruma, Feb 1891, *Menyhért 931* (holotype: Z; isotype: K!) • Changara, between Guro and Tete, 60 km from Tete, 1970, *M. A. Figueiredo 7* (WAG!) • Cabora Bassa, rectangular strip from the dam upstream, north slope (left bank) of Rio Zambeze, alt. 330–365 m, 1972, *Angelo Pereira & M. P. Correia 2031* (WAG!) • Zambezia Province: c. 60 km north-east of Mopeia Velha on the road to Quelimane, mixed deciduous woodland with *Albizia* spp., *Commiphora* sp. and *Strychnos* sp., climber with tendrils, flowers pale cream with yellow anthers, 8 Dec 1971, *G. Pope & T. Müller 540* (K!) • Mopeia, Campo, ao km 25, estrada para o régulo Raposo (Gh.), alt. c. 70 m, 27 Dec 1957, *A.R. Torre & M.F. Correia 16733* (K!). Tanzania. Morogoro Region • Kilosa District, Mikumi Division, Kisanga Ward, Kisanga Village, Ilole Forest, 07°25'27"S, 036°43'46"E, alt. 1723 m, 2007, *M. A. Mwangoka & J. Kidanka 5214* (WAG!). Malawi. Northern Region: Rumphi District, St. Patrick’s Mission, 1 mile E of Rumphi, alt. 3500 ft, 2 Apr 1977, J. Pawek 12572 (MO!). Southern Region • Mangochi District, T.A. Mponda, graveyard near Mangochi–Monkey Bay road, 24 Oct 1979, *A.A.K. Banda 1584* (MO!). Zambia. Central Province • Feira District, Feira Rd., 12 km S from Katondwe Mission, 15°20'S, 30°18'E, alt. 450 m, on edge of mopane woodland, herbaceous liana 3 m high, flowers pale orange-yellow, 1 Jan 1973, *Jan Kornas 2906* (K!). Zimbabwe. Sipolilo District • W of Kanyemba, mixed lowveld savanna woodland, twining plant, flowers pale yellow, alt. c. 550 m, 27 Nov 1966, *T. Müller 224* (K!).

### Key to the species of *Eureiandra* Hook.f.

The following key is adapted from the regional keys and species accounts in "Flora of Tropical East Africa" (Jeffrey 1967) and "Flora Zambesiaca" (Jeffrey 1978). Additional couplets were compiled from the species accounts in "Flora of Somalia" (Jeffrey and Thulin 1993) and Newton and Njoroge (2023).

**Table:** 

1	Leaves usually entire or shallowly 3 or 5 lobed (lobes less than halfway to the midrib); seeds compressed, minutely bidentate on both sides at the base; distributed only in Socotra Island	***E. balfourii***
–	Leaves usually deeply 3 or 5 lobed (lobes reaching about halfway to the midrib or deeper); seeds turgid, edentate at base, distributed in continental Africa	**2**
2	Plant from Ethiopia, Somalia or northern Kenya	**3**
–	Plant from West, Central, Northeast, East or South Tropical Africa, excluding Ethiopia, Somalia and northern Kenya	**4**
3	Male flowers solitary; pedicels 12–30 mm long; receptacle-tube 13–26 mm long; leaves 7–12 × 8.5–12 cm	***E. cogniauxii***
–	Male flowers in small fascicles; pedicels 4–12 mm long; receptacle-tube 10–11 mm long, densely pubescent; leaves 1.5–4.5 × 1.5–4 cm	***E. somalensis***
4	Stems nearly glabrous, sparsely hairy at the nodes; typically flowering with leaves	***E. orientalis***
–	Stems pubescent or hairy, or flowering when leafless	**5**
5	Anther locules densely covered with curved long hairs; bracts small, filiform	***E. lasiandra***
–	Anther locules glabrous or only minutely puberulous; bracts linear, elliptic to ovate-cordate, or inconspicuous	**6**
6	Bracts elliptic to broadly ovate-cordate, often lobed and leaf-like	***E. eburnea***
–	Bracts linear or inconspicuous	**7**
7	Corolla 7.5–15 cm across; receptacle-tube about 12 mm long	***E. formosa***
–	Corolla much smaller (petals 1.8–4.7 cm long); receptacle-tube 2.5–8.5 mm long	**8**
8	Stems perennial, softly woody; anther thecae glabrous; flowers pure white	***E. leucantha***
–	Stems annual, herbaceous; anther thecae minutely hairy; flowers deep cream to orange-yellow, rarely white	***E. fasciculata***

## Discussion

The present study confirms that the taxon previously treated as *Eureiandra* sp. A by Jeffrey (1967) is a distinct species and warrants formal recognition. Our material agrees well with the concept of that unnamed taxon and corresponds to the unpublished name *Eureiandra orientalis* Q. Luke (ined.) used later in the literature. The long interval between its first recognition and its formal description highlights the incomplete documentation of the plant diversity of East Africa (Holstein and Renner 2011). This pattern is further exemplified by a series of previously overlooked Cucurbitaceae species described from Kenya during the past decade, including *Peponium* and *Zehneria* (Zhou et al. 2016; Wei et al. 2017; Mutie et al. 2020; Watuma et al. 2019; Ngumbau et al. 2020b; Wei et al. 2020).

Morphological differences are stable across the available specimens and support the recognition of *E. orientalis* as a separate species. That said, the phylogenetic position of *E. orientalis* within the genus remains to be confirmed with molecular data. This underscores the broader need for molecular approaches to resolve phylogenetic relationships in understudied tropical lineages, where morphology alone may not fully capture evolutionary history (Holstein 2015; Bellot et al. 2020; Chomicki et al. 2020).

The discovery and formal publication of this species further emphasize the importance of the coastal forests of Kenya, Tanzania and northern Mozambique, a region widely recognized as a center of exceptional plant diversity and endemism that continues to reveal significant taxonomic novelties (Burgess et al. 1998; Timberlake et al. 2011; Darbyshire et al. 2020; Ngumbau et al. 2020a). Future studies integrating expanded field sampling, herbarium revision, and phylogenomic data will be important for clarifying species limits, relationships within *Eureiandra*, and the biogeographic significance of this eastern African coastal lineage. The currently available evidence supports recognition of the species on morphological grounds, but a broader reassessment of the genus would be valuable.

## Supplementary Material

XML Treatment for Eureiandra orientalis

## Additional information

### Artificial Intelligence (AI) use

The authors accept full responsibility for the content of the manuscript, including the disclosure of any use of AI.

No AI tools were used in the preparation of this manuscript.

### Author ORCIDs

R.-Z. Wang https://orcid.org/0009-0006-8710-7493

Z.-H. Ran https://orcid.org/0009-0000-5213-6936

H. Schaefer https://orcid.org/0000-0001-7231-3987

Q. Luke https://orcid.org/0000-0002-7256-031X

N.P. Mollel https://orcid.org/0000-0002-4402-4667

P.M. Kirika https://orcid.org/0000-0002-4318-9339

C. Chapano https://orcid.org/0000-0002-1932-5505

X. Yan https://orcid.org/0000-0003-4460-8117

S.-W. Wang https://orcid.org/0000-0002-8785-5065

N. Wei https://orcid.org/0000-0002-0313-3912

Q.-F. Wang https://orcid.org/0000-0001-9143-8849

### Data availability

All of the data that support the findings of this study are available in the main text.
